# Draft Genome Sequence of *Salinivibrio* sp. Strain EAGSL, a Biotechnologically Relevant Halophilic Microorganism

**DOI:** 10.1128/MRA.01020-20

**Published:** 2020-10-22

**Authors:** Erin M. Gaffney, Matteo Grattieri, Shelley D. Minteer

**Affiliations:** a Department of Chemistry, University of Utah, Salt Lake City, Utah, USA; University of Southern California

## Abstract

The halophilic bacterium *Salinivibrio* sp. strain EAGSL was isolated from the Great Salt Lake (Utah) for use in microbial electrochemical technologies experiencing fluctuating salt concentrations. Genome sequencing was performed with Ion Torrent technology, and the assembled genome reported here is 3,234,770 bp with a GC content of 49.41%.

## Abstract (precis)

The halophilic bacterium *Salinivibrio* sp. strain EAGSL was isolated from the Great Salt Lake (Utah) for use in microbial electrochemical technologies experiencing fluctuating salt concentrations. Genome sequencing was performed with Ion Torrent technology, and the assembled genome reported here is 3,234,770 bp, with a GC content of 49.41%.

## ANNOUNCEMENT

*Salinivibrio* sp. strain EAGSL is a Gram-negative, halotolerant, aerobic bacterium that was isolated from the Great Salt Lake (Utah) for its unique capability to establish electrical communication with an electrode surface (1). Microbial electrochemical technologies (METs) use this capability to employ bacteria as biocatalysts for distributed microgeneration/small-scale generation of energy, green electrosynthesis, and biosensing and are of particular interest for environmental applications due to their long-term stability, owing to the self-replicative nature of bacteria (2, 3). However, few bacterial strains are known to be fit for environmental METs, due to the requirements for both anodic respiration activity and tolerance of dynamic environmental conditions (4). As a result, despite several research efforts, reports of environmental METs remain relatively limited (5–9). Halophilic bacteria are appealing for microbial electrochemical treatment of harsh industrial wastewater (10), and *Salinivibrio* sp. strain EAGSL has shown self-sustained treatment of hypersaline wastewater containing 100 g liter^−1^ NaCl, allowing for understanding and development of METs capable of operation under environmental stress (1).

*Salinivibrio* sp. strain EAGSL was grown in growth medium for sulfate-reducing bacteria, due to reports of such bacteria in the Great Salt Lake, with the addition of 100 g liter^−1^ NaCl (11). The cells were then centrifuged for 20 min at 5,000 × *g*, and the pellet was used for genomic DNA (gDNA) extraction with a GenElute bacterial gDNA kit (NA2110; Millipore, Sigma). Whole-genome sequencing was performed by the DNA Sequencing Core Facility at the University of Utah using Ion Torrent technology (Thermo Fisher Scientific). One hundred nanograms of gDNA was used for library construction with the Ion Xpress fragment library kit (Thermo Fisher Scientific); library size selection (approximately 200 bp) was performed using the E-Gel system and SizeSelect 2% agarose gels (Thermo Fisher Scientific). Library size and concentration were confirmed with a Fragment Analyzer system using a high-sensitivity next-generation sequencing (NGS) kit (Agilent Technologies). The gDNA library was diluted to 23 pM and subjected to emulsion PCR using the Ion PI Hi-Q 200 template kit (Life Technologies). After enrichment, the final library was loaded onto an Ion PI chip and sequenced using the Ion Torrent Proton platform with Hi-Q sequencing chemistry. Reads were trimmed of adapter sequences and poor-quality regions by the Ion Torrent system, resulting in a total of 63,412,335 reads with a median length of 190 bp, giving an average read depth of 1,193×.

The resulting reads were assembled with SPAdes version 3.13.0 (12) (https://cab.spbu.ru/software/spades) using default settings, with the exception of using the iontorrent option. The assembly was checked using QUAST (13) (http://cab.cc.spbu.ru/quast), with gene prediction and annotation by the NCBI Prokaryotic Genome Annotation Pipeline (PGAP) feature with deposition. The resulting assembly contains 54 contigs, with an *N*_50_ value of 146,331 bp; the longest contig is 317,291 bp. The total genome assembly is 3.2 Mbp, with an average GC content of 49.41%, and has a total of 2,929 predicted coding sequences.

### Data availability.

The draft whole-genome sequence and raw data are available through the National Center for Biotechnology Information (NCBI) with genome accession number JABWMG000000000, BioProject number PRJNA630393, BioSample number SAMN14833495, and SRA accession number SRR12507111.
